# The Sustain and Spread Framework: strategies for sustaining and spreading nutrition care improvements in acute care based on thematic analysis from the More-2-Eat study

**DOI:** 10.1186/s12913-018-3748-8

**Published:** 2018-12-04

**Authors:** Celia Laur, Jack Bell, Renata Valaitis, Sumantra Ray, Heather Keller

**Affiliations:** 1 Faculty of Applied Health Sciences, University of Waterloo, Waterloo, Canada; 2 School of Human Movement and Nutrition Sciences, The University of Queensland and The Prince Charles Hospital, Chermside, Australia; 3 NNEdPro Global Centre for Nutrition and Health, St. John’s Innovation Centre, Cambridge, UK; 4 Schlegel-University of Waterloo Research Institute for Aging, University of Waterloo, Waterloo, Canada

**Keywords:** Nutrition, Sustainability, Spread, Implementation, Knowledge translation, Participatory research, Acute care, Hospital

## Abstract

**Background:**

Successful improvements in health care practice need to be sustained and spread to have maximum benefit. The rationale for embedding sustainability from the beginning of implementation is well recognized; however, strategies to sustain and spread successful initiatives are less clearly described. The aim of this study is to identify strategies used by hospital staff and management to sustain and spread successful nutrition care improvements in Canadian hospitals.

**Methods:**

The More-2-Eat project used participatory action research to improve nutrition care practices. Five hospital units in four Canadian provinces had one year to improve the detection, treatment, and monitoring of malnourished patients. Each hospital had a champion and interdisciplinary site implementation team to drive changes. After the year (2016) of implementing new practices, site visits were completed at each hospital to conduct key informant interviews (*n* = 45), small group discussions (4 groups; *n* = 10), and focus groups (FG) (11 FG; *n* = 71) (total *n* = 126) with staff and management to identify enablers and barriers to implementing and sustaining the initiative. A year after project completion (early 2018) another round of interviews (*n* = 12) were conducted to further understand sustaining and spreading the initiative to other units or hospitals. Verbatim transcription was completed for interviews. Thematic analysis of interview transcripts, FG notes, and context memos was completed.

**Results:**

After implementation, sites described a culture change with respect to nutrition care, where new activities were viewed as the expected norm and best practice. Strategies to sustain changes included: maintaining the new routine; building intrinsic motivation; continuing to collect and report data; and engaging new staff and management. Strategies to spread included: being responsive to opportunities; considering local context and readiness; and making it easy to spread. Strategies that supported both sustaining and spreading included: being and staying visible; and maintaining roles and supporting new champions.

**Conclusions:**

The More-2-Eat project led to a culture of nutrition care that encouraged lasting positive impact on patient care. Strategies to spread and sustain these improvements are summarized in the Sustain and Spread Framework, which has potential for use in other settings and implementation initiatives.

**Trial registration:**

Retrospectively registered ClinicalTrials.gov Identifier: NCT02800304, June 7, 2016.

## Background

In healthcare, there is increasing understanding of how to implement care improvements and a recognition that sustainability should be considered as a process from the beginning of implementation [1–4]. The need to implement and sustain improvements is particularly relevant for improving nutrition care practices in hospitals. One in three patients are at nutrition risk on admission to hospital, leading to increased mortality, length of stay, and risk of readmission among other negative outcomes [5–7]. Research has also demonstrated knowledge and implementation gaps in the identification and treatment of malnutrition in hospital [8, 9] and there is a need to sustain and spread improvements when they have a positive impact on patient outcomes and care.

Understanding is lacking regarding ways to sustain improvements, however definitions of sustainability are said to have five key elements: *1) after a defined period of time 2) a program, clinical intervention, and/or implementation strategies continue to be delivered and/or 3) individual behavior change (*i.e.*, clinician, patient) is maintained; 4) the program and individual behavior change may evolve or adapt while 5) continuing to produce benefits for individuals/systems* [2]*.* Sustainability frameworks, such as the Dynamic Sustainability Framework, also acknowledge a constantly evolving context [10]. However, less is known about specific strategies to sustain and spread improvements once they have demonstrated initial success [3]. If other local teams or units could benefit from a successful change, “spread” is encouraged. Spread is defined as making localized changes along a specific care pathway, beyond the initial implementation location [11]. Only some of the learning from the initial implementation may apply when spreading to a new location due to differences in context, culture, and other factors. Consequently, re-working through each stage of implementation, such as following the Knowledge-to-Action framework, is recommended [1, 12]. Some spread may occur naturally, such as through sharing ideas with other staff [1], but this is not a guaranteed approach to spread. As with sustainability, little is known regarding strategies for spreading change. Sustaining and spreading changes is thought to lead to a culture change, which for purposes here is defined, but not limited to, shared beliefs, values, norms and routines [13, 14].

To address the gaps in hospital nutrition care with the aim of sustaining and spreading success, the More-2-Eat project used participatory action research to support and evaluate the implementation of the Integrated Nutrition Pathway for Acute Care (INPAC) [15], a ‘best practice’ pathway for improving nutrition care [9]. To determine the anticipated barriers and enablers to INPAC implementation, key informant interviews (KI) and focus groups (FG) were conducted with hospital staff and management before implementation (late 2015) [16], identifying five themes: building a reason to change; involving relevant people in the change process; embedding change into current practice; accounting for climate; and building strong relationships within the hospital team [16]. The aim of this manuscript is to develop a potential framework of strategies to sustain and spread the successful implementation of INPAC.

## Methods

### The More-2-Eat project

The More-2-Eat project facilitated implementation of INPAC, an evidence and expert consensus based ‘best practice’ pathway, in a single medical unit in each of five Canadian hospitals in four provinces. The size of the hospitals ranged from 186 to 1100 beds, with the unit size ranging from 27 to 50 beds. Further details of the More-2-Eat project, the multi-method data collection, and the hospital characteristics are available elsewhere [15, 17]. Participatory action research was used to encourage sustainable change; local champions were encouraged to continue to lead and implement further changes, including spread, after project completion [18]. The Knowledge-to-Action framework [12], the Theoretical Domains Framework [19], the Consolidated Framework for Implementation Research [20], the Model for Improvement [21], and the Normalization Process Theory [22, 23], were used to support implementation of INPAC [15].

In the More-2-Eat project, each hospital unit had a “champion,” research associate(s), and an interdisciplinary site implementation team that planned the best practice activities to implement and integrate into the unit routine. Each unit had one year (Jan-Dec 2016) for implementation; collection of INPAC audit data was reported back regularly to sites [15]. A community of practice (external researchers and facilitation team) supported champions via monthly telephone calls and used a listserv/email group for questions between meetings. Training for champions and site teams included change management, quality improvement, and behavior change, particularly the Michie et al., Behaviour Change Wheel, recognizing that Capability, Opportunity and Motivation (COM-B) was required to change team behaviour towards best practice [24].

### Sampling and recruitment

Two-day site visits for KI, FG and small group discussions were conducted in October/November 2016, after implementation. A minimum of 2 FG (4–10 people/group) and 6 interviews were conducted per site; 7 interviews were conducted by phone for participants unavailable during the visit. Purposive sampling was used for interviews so that valuable insight, both positive and negative, could be elicited; interviews were conducted with champions and research associates, as well as other key team members. All staff on the unit were invited to the FG by the champion or research associates using posters, e-mails, verbal encouragement, and enticement of a free lunch. Small group discussions (2–3 people per group) occurred when FG attendance was limited, or when those invited for individual interviews requested joint interviews. Although similar ideas continued to arise in the discussions after the third site visit, all prearranged KI/FG were completed to provide context specific data. To saturate developing themes on sustainability and spread of INPAC, in January/February 2018, a year after project completion, another round of KI and small group discussions were conducted by telephone with a More-2-Eat project champion and a purposively selected member of the site implementation team from each hospital.

### Data collection

CL conducted all KI and FG to allow for increased depth as she had conducted baseline interviews (Fall 2015) and understood the context [16]. CL is a female researcher in health studies, with a background in public health nutrition and implementation science and practice. She is not a health professional and not associated with any of the hospitals, although she did support the units to implement INPAC.

All FG occurred around lunchtime, and the environment was made to feel informal; participants could leave at any time as clinical commitments took priority. Written consent was complemented with verbal reminders about the recording and the purpose of the discussion. A More-2-Eat project champion or research associate took notes during the FG and this was explained to the group and included in consent. Discussions used an active interviewing approach and were based on a semi-structured interview guide (Table 1) adapted by CL for the profession and role [25]. KI and FG took between 15 and 75 min and were digitally recorded. Context memos for all KI, FG, and sites elaborated on key observations and reflections. Sites were given a brief summary with key considerations after the site visit and again after the sustainability interviews. As a form of member checking, each site could respond to the summaries if they felt it was inaccurate. Verbatim transcription was completed by a professional service for all interviews. FG recordings were not sent for transcription due to the volume of KI data. As a result, FG data were considered complementary in analysis. Key points and quotes from each FG were obtained by listening to recordings a minimum of twice (CL). To interpret the participant codes in the results, I = End of Implementation Phase and S = Sustainability Phase; and sites (hospitals) are labeled as A, B, C, D, E. When more than one profession is provided, this indicates one person holding two roles (i.e. Nurse + Manager).

**Table 1:** Interview guide for post-implementation and sustainability interview

Post-Implementation Interview Questions	Sustainability Interview Questions
What changes happened over the past year and did it impact: Your practice? The practice of your staff? - What was done day-to-day? - The norm of care on the unit? If no change noticed, why not? What was the impact of these changes on patient care? What and who supported these changes? How? What else would you like to change? Did you receive any nutrition education, and if so, when, what type, delivered by whom? What were the main factors that influenced implementation? What could have been done differently to improve nutrition care? How do you plan to sustain the successful changes? What should be included in a toolkit to help other hospitals starting to improve nutrition care? Do you have any advice for other hospitals starting to improve nutrition care?	Do you think nutrition care is still important on the study unit? In the hospital? Do you think the changes made to improve patient care are part of the routine? How do you know? How did you encourage them to be part of the routine? What happened to the implementation team after More-2-Eat ended? What was the impact? What strategies did you use to maintain focus on nutrition after the year of improvements? Which strategies were effective? Not effective? Did anyone continue to collect data to monitor progress after the end of official data collection? If so, what did you collect? How? Who saw the results? Did you take advantage of any new or existing opportunities to spread nutrition throughout your hospital? Do you think the champion role was sustained? How did it change? How did you continue to engage with stakeholders? What are your goals and next steps? Do you have any advice for other hospitals starting to improve nutrition care?

Note: not all questions were asked of all participants

### Analysis

**Fig. 1:**
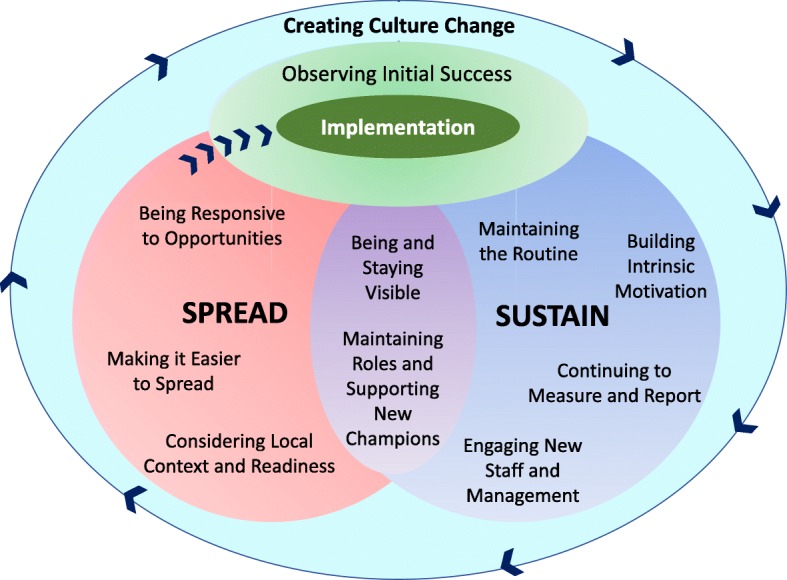
The Sustain and Spread Framework: Once there is initial implementation success, strategies are used to sustain and spread the successful change. Strategies to encourage changes to be sustained or spread are included within each circle, with the two strategies in the middle applying to both sustaining and spreading success. To fully spread into a new setting or unit, a new change goes back to implementation (arrows from the Spread circle back to Implementation) in the new context. Working through several rounds of sustaining and spreading may lead to an overall culture change

CL conducted initial analysis of interview transcripts, FG notes, and context memos using NVivo 11. The Saldana et al., inductive approach of first and second cycle coding was used, with one idea per first level “code” [26]. Second level codes were formed by grouping similar first level codes. Post-implementation interviews were analyzed first, and after a review of initial themes and transcripts (*n* = 12 transcripts; 4 per person; reviewed by HK, RV, and JB), it was decided that a final set of interviews a year after project completion (2018) would allow for saturation of themes on sustainability and spread. After line by line coding of these final interviews, thematic analysis was conducted combining both sets of results, and a framework created (Fig. 1). These results were shared with authors to check against additional transcripts (*n* = 8 2018 transcripts; 2 per person). Triangulation with other findings, including More-2-Eat project data and researcher memos were also used to confirm the themes [15, 17, 27].

## Results

A total of *n* = 138 participants were involved (Table 2; Note sustainiability participants were also participants in the post implementation phase). Results suggest that several implementation changes were sustained and spread successfully leading to an overall culture change whereby the importance of nutrition care to the recovery of patients was prioritized. Successful implementation included improving processes, perceptions, and ultimately patient outcomes, as described elsewhere [28, 29]. Based on this success observed by sites, focus shifted to strategies to sustain and spread improvements, which also provided opportunities for implementation of further best practices. One small change was unlikely to lead to a culture change, but a series of changes that were sustained and spread did result in a shift in values towards the importance of nutrition care as indicated in the framework (Fig. 1).

**Table 2:** Participant demographics

Demographic Information		Post Implementation Phase			Sustainability Phase
		Interviews *n* (%)	Small Group Discussions (≤3 people); n (%)	Focus Groups (4+ people); n (%)	Interviews n (%) + Small Group Discussion^a^
# of Participants		45	10	71	12
Gender	Female	40 (89%)	6 (60%)	61 (86%)	10 (83%)
	Male	5 (11%)	4 (40%)	9 (13%)	2 (17%)
	Missing Data	0	0	1 (1%)	0
Age Group	< 30 years	3 (7%)	2 (20%)	19 (27%)	0
	30–39 years	10 (22%)	2 (20%)	21 (30%)	1 (8%)
	40–49 years	14 (31%)	3 (30%)	13 (18%)	3 (25%)
	50–59 years	13 (29%)	3 (30%)	13 (18%)	5 (42%)
	60+ years	3 (7%)	0	4 (6%)	0
	Prefer not to say	1 (2%)	0	0	1 (8%)
	Missing	1 (2%)	0	1 (1%)	2 (17%)
Profession	Dietitian	16	2	6	5
	Diet Technician/Diet Assistant	1	0	2	0
	Food Service Supervisor/Manager	7	1	0	1
	Registered Nurse	9	4	25	2
	Registered Practical Nurse/Licensed Practical Nurse	1	2	7	0
	Health Care Aide/Personal Support Worker	0	0	5	0
	Attending Physician	2	1	4	1
	Physiotherapist/Occupational Therapist	0	0	9	0
	Pharmacist	0	0	3	0
	Management	14	2	0	7
	Other^b^	2	0	10	1
	Missing	1	0	0	0

^a^Small group discussion was *n* = 2;

^b^Other: researcher, rehabilitation, volunteer coordinator, clinical care lead, administration support, food service worker, nurse educator, discharge planner, Speech-Language Pathologist

Note: some participants indicated more than one profession, therefore the profession values will not equate to the total number of participants

### Sustain

All sites experienced a shift from implementation to sustainability. “*With any initiative, the most difficult piece isn’t the processes themselves. It’s the change management and sustaining those improvements.”* (IA-14:Nurse). Specific strategies to sustain a change included: maintaining the new routine, building intrinsic motivation, continuing to measure and report, and engaging new staff and management.

#### Maintaining the new routine

After a change had started becoming embedded into the routine, sites recognized that effort was still needed to keep it going. “*We have to build it, and then we still have to maintain it and then we’ll see the effects.”* (IA-11:Registered Dietitian [RD]). Sites also had to make sure the change was having the desired effect. *“Making sure that what we’ve set up is actually working. ... You can’t just put something in place and hope that it’ll continue to run successfully.”* (SC-1:RD + Manager).

To maintain the new routine, key unit staff needed to remain involved in keeping others engaged. *“In addition to a clinical manager, we have nurse educators and clinical care leaders; those are key because they’re the ones that are going to be continuing to talk and have the discussions around nutrition care in the absence of clinical nutrition.”* (SB-1:RD + Manager). Supportive unit managers and nurse educators were key to delivering education, answering questions, and providing continued support, reminders and progress updates after the implementation team moved on to other priorities for improvement: “*If you see something falling by the wayside, keep subtly putting it in there again.”* (ID-5:RD).

The change also had to be seen as part of the job, building accountability, such as through performance reviews, or finding ways to standardize the process (e.g., Standard Operating Procedures etc.). Maintaining the new routine was about making sure staff had what they needed and it was easy. *“I think just making sure that you give them the tools. I mean, they’ll do it if it’s easy and if it’s there.”* (SB-2:Manager).

#### Engaging new staff and management

Participants discussed the challenges of high staff turnover. “*It’s that maintenance and continuing to collaborate with new staff that are coming onboard, be it frontline nurses or new managers or new volunteer coordinators or physicians. It’s a continuous need to remind and raise that awareness.*” (SC-1:RD + manager). For engaging new management it was about giving them some time to understand the new environment and then setting up a brief meeting to explain that nutrition care was prioritized, what had been achieved, and future plans. “*You have to give people a bit of time to kind of get acclimatized to the unit* [and then bring them on board]” (SE-2:RD + Manager). One champion who experienced high turnover, indicated: *“A lot of my work has just been making sure that things carry on with new leaders.”* (SC-1:RD + Manager).

For engaging new staff, integrating key messages into the orientation was a key strategy. “*We also mention it* [nutrition] *at new hire orientation*.” (SC-2:Nurse Educator). In this way, nutrition care was not seen as “new,” and could be treated as a valued and expected practice on the unit. Nurse educators also supported new staff. “O*ur educator, who was part of our team, is doing another round of education and awareness-building because of the* [staff] *turnover.*” (SC-1:RD + Manager).

#### Building intrinsic motivation

Intrinsic motivation, as noted in the baseline analysis theme of “building a reason to change” [16], is needed to undertake and sustain a change in practice. Those who work in healthcare typically have the intrinsic motivation to help their patients; recognizing that improving nutrition care enhances patient recovery supports this intrinsic motivation.

“*99% of the people, or higher, who work here want to help people and want to help the patients. So, I think we need to start with making sure that the managers buy in and see that this is really worthwhile doing, and getting some key front line champions onboard that say, ‘Yeah, I think this is important. … I’d like to do it, but I don’t want to be the only one doing it, so I’ll encourage my colleagues to do it.’”* (IB-4:Manager).

Recognizing that everyone has a role to play in improving nutrition care for patient benefit was also demonstrated as a way to build intrinsic motivation. Staff were able to see their specific role, and the impact they could have. “*We also developed a tool called “Find, Feed, Follow,” and it’s for every discipline. Our Malnutrition Steering Committee discussed each in an interdisciplinary group, and they each kind of discussed what their role is to find people with malnutrition, to feed them, and to follow. There was a lot of “a-ha” moments with the team, realizing that, “Oh, a piece connects to my world.” So, that was valuable.”* (SE-1:Manager).

Intrinsic motivation was also built by engaging staff throughout the change, including involving them in decisions. *“I think really asking nursing and staff feedback was a good way to start and a good way to continue on through. I think it kept them engaged.”* (ID-1:RD). Encouragement when staff were doing well also facilitated continued motivation. *“Recognizing staff for the work that they’re doing. When they hear, “This is really good work. Keep it up,” … It starts to become more of an intrinsic motivation to do it,* versus*, “We’re doing this because we have to.””* (IA-12:Nurse). However, with busy hospital staff, intrinsic motivation on its own may not be not enough: “*We still have resistance from nursing. I say that, being one. Can’t get them to prioritize patient setup or even bedside table setup for us. We’ve struggled with that. They have multiple competing priorities and will tell you that’s not their first priority. …We don’t feel that that’s as important as it is.*” (IB-9:Manager). Although intrinsic motivation is important, it may not be sufficient to sustain a change.

#### Continue to measure and report

Data was seen as essential for implementation and sustainability. *“It* [data] *needs to be local, it needs to be timely and it needs to be in a format where you can see your trend and your results. The reinforcement is extremely important.”* (IA-15:Manager). A strong implementation plan should include collection of data from baseline, throughout implementation, and continue longer term to show if the changes were being sustained. Monthly INPAC audit reports based on chart review completed twice per month served this purpose in More-2-Eat. “*We have to keep auditing. Audits are a huge thing. If you keep auditing and you see that it’s fallen to the wayside then you can talk about it more and keep trying to sustain everything that we’ve started.”* (ID-4:RD + Manager). Audits may not need to be collected as regularly as during implementation, however they are still important. *“There needs to be dedicated audits. We need to see where the gaps are. … I don’t think it needs to be at the same frequency, but I think that it’s important that we maintain that for momentum.”* (IC-4:Nurse+Manager).

Reporting results were also key for sustainability, engagement, and contributed to building intrinsic motivation. *“They can take pride in it* [audit results]*, and then, therefore, I think it’s just intrinsically rewarding themselves. And they go, “Well, I’m going to keep doing this because, look at that, I get this…” they get the feedback about it.”* (IA-10:Manager). Specific strategies for relaying audit results included huddles, quick chats with individual staff, e-mails, posters etc. “*The audits were the most important thing. Then when they were noticing a dip down in practice, then we would talk about it at huddles. I would send out emails and let them know what the compliance was and that they needed to improve that.*” (IC-4:Nurse+Manager). Audit results were also useful for management. *“I always want to see results. I want to see, ok, we’re doing this study, we’re doing it, but I want to see results, and that it’s working. … What it improved… How?... Show me the numbers…”* (ID-FG1).

### Spread

When a change was seen as having a positive impact, it led to the desire for other units and hospitals to consider that improvement. *“I’ve been happy with how it’s starting to seep out to other areas within the organization*.” (IB-8:RD + Manager). A year after project completion, nutrition screening and use of a standardized assessment to diagnose malnutrition (i.e., subjective global assessment; SGA) were used hospital-wide in all sites and had also spread to other local hospitals. Strategies for spreading successful changes included: being responsive to opportunities, considering local context and readiness, and making it easy to spread.

#### Being responsive to opportunities

Other units and hospitals were interested in implementing the successful changes from the pilot units. “*We are still hearing from other units… They’re asking us, ‘When are you going to roll out that form on our unit,’ or, ‘When are you going to roll out that initiative on our unit?’ So, there still is interest out there.”* (SE-2:RD + Manager). Recognizing that interest, responding, and providing support helped spread and maintain the momentum. *“If the interest or the desire is there, I think what we have to do is kind of capitalize on when that interest is being expressed.”* (IB-8:RD + Manager).

These opportunities could arise from the micro (individuals, unit etc.), meso (hospital etc.) or macro (regional etc.) levels, and each could be utilized in their own way. At the micro level, individual interest could spur change: *“If there’s an interest, they volunteer, than they’ve already met half of the battle by demonstrating their interest.”* (SB-1:RD + Manager). An example of a meso-level opportunity was leadership demonstrating their support for food and nutrition initiatives. *“This idea of having the executive team deliver meal trays when they’re doing rounding with patients would be, I think, a good way to get staff to buy in that, you know, it’s not just your responsibility, but we’re all kind of doing our part.”* (SE-2:RD + Manager). At the macro level, aligning the regional initiatives provided many opportunities such as materials, resources, and benefits of having similar goals. *“Our healthcare region has probably been the strongest impetus moving things forward.”* (IE-2:RD + Manager). Examples of opportunities being seized included when admission forms were being changed anyways, *“they wouldn’t change the forms for us unless they were already being changed*” (IB-4:RD); when a new electronic medical record systems was being set up; or when nutrition could connect to another priority, such as patient safety.

#### Considering local context and readiness

Each unit was unique so local context and readiness needed to be determined before starting full implementation. *“How others should do it has to be driven by what makes the most sense on those units.”* (IB-8:RD + Manager). An individualized approach to spread was encouraged. *“I would view it as a unit-by-unit implementation. … healthcare has its own culture, and change is difficult… you need to sort of make sure that everybody buys in. I’ve seen far too many projects where we try to do this wholesale implementation, and they fail. So, I think it’s much better to do it smaller scale and slow steps, and then, before you know it, it’s replicated across the patch and you don’t have to worry about selling people because it sells itself.”* (IA-6:Manager).

Checking for unit readiness was about understanding what was happening to see if it was the right time to encourage implementation in specific units. *“To take a look at if there’s readiness. I’d like to be able to promote some of the results that will come forth in the upcoming months and years to really start understanding with units as to who’s ready, who might like to look at an implementation, and who might like to take a look at making some changes.”* (ID-6:Manager).

Units that have expressed interest and had a strong team seemed to be the ones who were ready for implementation. *“We find that if singular units have readiness and they have a cohesive team and want to work together and do more, these would be some of the ways that we could approach it and take a look at trying to implement some of the same things and using the tools that were already created to help.”* (ID-6:Manager).

#### Making it easy to spread

After learning from initial implementation, several meso and macro level changes were used to make it easier for the change to spread. One aspect was understanding the barriers that were faced in initial implementation, and being upfront and working to overcome these earlier in the new setting: “*Just being open and honest and telling them that these are the obstacles that we’re going to come across.”* (SE-1:Manager).

Having systems already set up also made it easier for new units. For example, when the screening and referral process was already in the computer system, the focus was on changing behavior so the system was used, rather than setting-up the system. Examples included having screening questions embedded in forms or the malnutrition assessment components “w*e actually embedded it* [subjective global assessment] *into our initial nutrition assessment documentation form. That definitely… oh, yeah, that makes a difference.*” (SE-1:Manager); setting up medication pass (oral nutritional supplementation delivered with medication) with an already available product; or including the best practices in standard operating procedures.

Learning from each other also made it easier. *“I wouldn’t mind just being part of helping other units start all of this – like, a little bit of hand-holding because sometimes I find that people need that.”* (IB-I1:RD). Another manager indicated the benefits of learning from past experience: *“Whenever I’m looking to roll something new out, I don’t want to reinvent the wheel. I want to go to somebody that’s tried and true, and steal shamelessly from them and use what I can.”* (IA-6:Manager).

### Connecting spread to implementation

To spread a change so it was fully embedded into practice and could be sustained, the implementation process started again in each new location and for each new improvement activity. In Fig. 1, this theme is represented by the arrows from *Spread* to *Implementation*. When asked how a change should be rolled out to other units, a participant replied: *“The exact same way as you guys did on this unit. Introducing it to the staff. Making it part of the admission process. Increase awareness.”* (ID-FG2). When spread happened without considering the full change management process, there were more challenges. *“We’ve gone ahead with MedPass and with screening and SGA and discharge planning* [on a new unit]*, but it needs to be heightened. We’re not getting the referrals, and we’re pretty sure that the screening isn’t being done well. So, it’ll be kind of going back to square one and doing more of that team approach and seeing what we could do to influence that.”* (SC-1:RD + Manager). Another participant learned that full implementation was needed in a new location. “*We should’ve gone to the front line staff in the first place, got them to help us build it. … We just assumed that it would be pretty plug and play. It turned out not to be. So, if I was to do it again, I would’ve gotten the front line staff to help us.”* (IB-9:Nurse+Manager).

### Sustain and spread

Both sustaining and spreading a nutrition activity required two further strategies, being and staying visible and maintaining roles and supporting new champions.

#### Being and staying visible

Being able to see the change and the people driving it were important for both sustaining and spreading changes. *“I’m just wondering if that presence and visibility* [of the project and dietitian] *has helped to kind of sustain the changes more so than something more specific, like an education session or an auditing process.”* (SB-1:RD). The change had to stay visible so people would keep talking about it, thus encouraging it to become embedded. *“I think part of it is just through osmosis, right? Like, we talk about it so much, and we do things so much. And sometimes some of the front line staff won’t put two and two together that the osmosis is from us talking about it. But I think that’s when the real benefit is, is that people start just naturally putting things into their day-to-day practice.”* (IB-7:Manager). For a nutrition focused project, visibility included having dietitians on the unit regularly, available for questions, and continuing to talk about success. *“We’re in their faces all the time. We’re on the units all the time.”* (IC-2:RD).

#### Maintaining roles and supporting new champions

A champion was needed throughout implementation and to sustain and spread changes with the support of an implementation team: *“Somebody has to own it. Because if nobody owns it, then it goes by the wayside.”* (IA-11:RD). This champion also supported others to champion specific changes or areas to spread. *“We have a lot of people here who are very good at driving change and driving initiatives that are specific to this unit. I think that it will be important to take those people as champions.”* (IC-4:Nurse+Manager). Involving existing leaders, including those who are seen as leaders by other staff, helped with buy-in and to drive a change. *“You need to get that buy in and you need to scout out who are your leaders or who has more input with the staff or who are the staff who’s kind of their champion... Then make sure that those people are involved as well.”* (SC-2:Nurse Educator).

After initial changes were in place, the implementation team either stayed in place, shrank, or merged into existing teams, such as those focused on changing practice or quality improvement. *“I think our Quality Council is the place to be and the place to bring up what changes you think need to happen and then work on a plan.”* (SD-1:RD). Regardless, developing and maintaining champions was a required strategy to spread or sustain improved nutrition practices.

### Creating culture change

While sustaining and spreading successful improvements, a culture change was discussed by participants. *“People are thinking about it, know about it, feel it. There’s a presence there, and so that’s maybe a start to a change in nutrition culture.”* (IB-8:RD + Manager). People were paying attention to the changes and their impact, particularly for their benefit to the patients. *“I think it’s made a big difference. I think hopefully we’re preventing people from being readmitted. I think we’re seeing the people that we really need to see… I think it’s really, really helped improve our patient care.”* (IC-2:RD). This reported culture change was visible in a variety of ways such as: *“people are paying more attention to what people* [patients] *are eating.”* (IE-FG1); *“we are more aware of it as a group, particularly the physicians.”* (IA-FG2:Physician); *“myself and my staff have become more aware of malnutrition as an issue. Conversation comes up more frequently during our discharge rounds and just day-to-day time on the unit. We discuss food much more.”* (IB-7:Manager).

Culture change within administrators was also demonstrated through change in allocation of resources. In one site, it was originally mentioned that *“budgets being so tight, there’s no appetite for any investment at all*” (IA-6:Manager). After the More-2-Eat project ended, a request to continue specific nutrition care processes was approved. Dedicated resources to facilitate champion time was also seen as beneficial. “*The real key, honestly, is being able to have some dedicated resources to continue to follow up and observe and audit and review and look for continuous ideas as to how to improve and engage improvement specialists for you to support that message. The challenge is, however, that resource isn’t readily available.*” (ID-2:Manager). In the More-2-Eat project, a year after the small influx of resources to support data collection and champion time had ended, 2018 interviews indicated most changes had been sustained and spread. “[Nutrition care] *continues to be a culture within our study unit.*” (SB-1:RD + Manager). Changes had become “*embedded into our routines and our relationships*” (SA-3:Manager). It was clear that even though these changes had started as part of a research project, the end of the project did not indicate the end of nutrition care improvements. “*I don’t think this thing is ever going to end to be honest... I think this is just a start, and then after the study’s over, we need to continue. That is something that speaks to me loud and clear, that this isn’t something that just stops after the study’s over. We’ve got to keep going and figuring out how we can continue making it important, that nutrition is important, and that food is medicine.”* (IC-1:RD). It is apparent that successful implementation, sustaining and spread could lead to what was described as a culture change.

## Discussion

This analysis, although specific to the context of improving nutrition care in hospitals, resulted in the Sustain and Spread Framework (Fig. 1), which may be applicable to other healthcare implementation initiatives. This framework may be used as a guide for other quality improvement initiatives or policy changes after initial success with implementation, so changes are sustained and spread.

The “implementation” circle of the Sustain and Spread Framework could include any existing framework, including those presented in baseline results [16], as well as the Knowledge-to-Action framework [12], the Model for Improvement [21], the Normalization Process Theory [22, 23], or any other model of implementation. These are also the models on which the More-2-Eat project is based [15]. Within implementation and throughout sustain and spread are the overarching principles of the Theoretical Domains Framework [19], the basis for the Behaviour Change Wheel [24], that lists interventions and techniques to create change at various levels of influence. The More-2-Eat project champions and research associates were trained on the Behaviour Change Wheel, and results indicate that a variety of strategies were used to change behaviour [30].

The More-2-Eat project is in line with Organizational Participatory Research, in which organizational changes and practice improvements are made [31]. Within Organizational Participatory Research, additional benefits exist that are likely to contribute to sustained change and improved adoption of future changes, as this style of research can empower healthcare professionals and improve their career development, benefitting the individual and organization [31]. Overall, champions and their teams are key to implementing and sustaining change, which literature also suggests [4, 13, 32–34].

### Making lasting improvements to nutrition care

Improvements in hospital nutrition care were driven by a series of related changes that were sustained and spread using overlapping strategies. A strong foundation of implementation led to initial success and then shifted into sustaining and spreading those changes. After determining readiness for spread, implementation started again in new areas, continuing to change staff values. Keeping the initiative visible and having champions maintain their roles and support new champions was essential. These results are in line with other sustainability literature that suggests organizational factors, funding, support (e.g., champions), and practitioner characteristics (e.g., turnover) are particularly relevant [4].

With all of these elements in place, some sites started to recognize organisational culture change. The reported consistencies across definitions [13, 14] were seen throughout the project, including shared beliefs, values, norms and routines among the staff on the More-2-Eat units. As participants reported a shift in the way people throughout the organization thought about nutrition, responded to malnourished patients, and adapted their practices, results were in line with the thinking that changing core values can help shift institutional culture [35].

### Being flexible

Some sustainability literature describes the tension that can arise between having a change become embedded into routine, while still allowing for future innovations [1, 4]. Sustainability was seen by the More-2-Eat project units as a process, recognizing that even once change is embedded, refinement with implementation cycles are still needed to keep the change going [1, 21, 36]. Sites allowed for new opportunities or changes to existing processes, yet surprisingly, there was little mention of removing processes that were not working or low value (de-implementation) [37, 38].

Some staff requested a “reprieve” from change, as they wanted a chance to get accustomed to a new process before the next change started. Other staff recognized the hospital environment needs to be continually adapting to best meet patient needs, and requested more “refreshers” to make sure nothing was forgotten. Flexibility within implementation was also considered important to accommodate the busy clinical environment and encourage adoption [4, 20]. Following the More-2-Eat project, INPAC was adapted to be less prescriptive to encourage this flexibility [39] and More-2-Eat Phase 2 is testing a sustainable model to encourage further spread. To guide anyone interested in making nutrition care improvements, the INPAC implementation virtual toolkit was developed. The toolkit provides specific direction for making improvements and includes key messages, quotes, videos, resources and tools to support implementation of INPAC [40].

### Strengths and limitations

Particularly in the 2018 interviews, champions or those that had been involved from the beginning were selected for participation based on their ability to reflect on the full process. Since they were intimately involved, in depth interviews were conducted, however their views may not be reflective of others on the unit, including patients and care partners, nor do interviews necessarily reflect the regional perspective. As KI and FG were arranged during two day site visits conducted across Canada, it was not possible to recruit participants only until saturation. As similar themes were seen after the third site, saturation was being approached, however all scheduled interviews were conducted to provide context specific data and increased depth of understanding. Having all data collection and analysis conducted by one researcher was seen as beneficial to encourage continuity across interviews and analysis. Addition of a second analyst may have been beneficial, however other authors reviewed a selection of transcripts, potential themes were discussed and several iterations of the diagram were reviewed throughout analysis.

“Scale,” “Scaling-up” or “Scaling out” are terms typically associated with sustain and spread, implying another approach to increasing the uptake of a change [1, 41, 42]. These terms are not used in this framework as they focus on broader, top-down implementation that is leader-heavy, thus not representative of the process discussed by participants [43].

Units, which were selected based on their readiness to change, were provided with a small financial incentive (mainly for data collection [15]), and received coaching from a research team, all factors that would not typically be available to a hospital. As the idea of culture change was not considered before analysis, questions were not developed with consideration of culture change principles or theories.

## Conclusion

This study revealed key strategies used to sustain and spread successful changes. Although based on nutrition care improvements, these strategies have been summarized in the Sustain and Spread Framework, which may be useful in other healthcare implementation initiatives. This framework has potential to strengthen the way successful changes are sustained and spread to allow for longer term improvement in patient outcomes.

## Abbreviations

FG: Focus Group
INPAC: Integrated Nutrition Pathway for Acute Care
KI: Key Informant
RD: Registered Dietitian
